# Efficacy of Genetically Modified Bt Toxins Alone and in Combinations Against Pink Bollworm Resistant to Cry1Ac and Cry2Ab

**DOI:** 10.1371/journal.pone.0080496

**Published:** 2013-11-07

**Authors:** Bruce E. Tabashnik, Jeffrey A. Fabrick, Gopalan C. Unnithan, Alex J. Yelich, Luke Masson, Jie Zhang, Alejandra Bravo, Mario Soberón

**Affiliations:** 1 Department of Entomology, University of Arizona, Tucson, Arizona, United States of America; 2 USDA-ARS, U.S. Arid Land Agricultural Research Center, Maricopa, Arizona, United States of America; 3 National Research Council of Canada, Biotechnology Research Institute, Montreal,Quebec, Canada; 4 State Key Laboratory for Biology of Plant Diseases and Insect Pests, Institute of Plant Protection, Chinese Academy of Agricultural Sciences, Beijing, People's Republic of China; 5 Instituto de Biotecnología, Universidad Nacional Autónoma de México, Cuernavaca, Morelos, Mexico; University of Tennessee, United States of America

## Abstract

Evolution of resistance in pests threatens the long-term efficacy of insecticidal proteins from *Bacillus thuringiensis* (Bt) used in sprays and transgenic crops. Previous work showed that genetically modified Bt toxins Cry1AbMod and Cry1AcMod effectively countered resistance to native Bt toxins Cry1Ab and Cry1Ac in some pests, including pink bollworm (*Pectinophora gossypiella*). Here we report that Cry1AbMod and Cry1AcMod were also effective against a laboratory-selected strain of pink bollworm resistant to Cry2Ab as well as to Cry1Ab and Cry1Ac. Resistance ratios based on the concentration of toxin killing 50% of larvae for the resistant strain relative to a susceptible strain were 210 for Cry2Ab, 270 for Cry1Ab, and 310 for Cry1Ac, but only 1.6 for Cry1AbMod and 2.1 for Cry1AcMod. To evaluate the interactions among toxins, we tested combinations of Cry1AbMod, Cry1Ac, and Cry2Ab. For both the resistant and susceptible strains, the net results across all concentrations tested showed slight but significant synergism between Cry1AbMod and Cry2Ab, whereas the other combinations of toxins did not show consistent synergism or antagonism. The results suggest that the modified toxins might be useful for controlling populations of pink bollworm resistant to Cry1Ac, Cry2Ab, or both.

## Introduction

 The insecticidal proteins of *Bacillus thuringiensis* (Bt) kill some major insect pests, but are harmless to vertebrates and most other organisms [1-3]. In 2012, farmers planted genetically engineered corn and cotton producing Bt toxins on 70 million hectares worldwide [4]. The most widely used Bt proteins are crystalline (Cry) toxins, particularly three toxins that kill lepidopteran larvae: Cry1Ab in Bt corn, Cry1Ac in Bt cotton, and Cry2Ab in second-generation Bt corn and Bt cotton [2,5,6]. Extensive use of Bt toxins in sprays and transgenic crops has caused field-evolved resistance in some pests, which entails a genetically based decrease in susceptibility [5-8]. Field-evolved resistance associated with reduced efficacy of Bt toxins has been reported in seven pest species, two targeted by Bt sprays [9,10] and five targeted by Bt crops [11-16]. Other cases of significant decreases in susceptibility to the Bt toxins in transgenic crops including "incipient resistance" and "early warning" of resistance have been detected in at least four additional pest species [8,17-21].

 One approach to counter resistance is to engineer Bt toxins to make them more effective against pests that are resistant to previously deployed toxins. For example, the genetically modified Bt toxins Cry1AbMod and Cry1AcMod were effective in laboratory diet bioassays against some strains of six species of Lepidoptera that are resistant to the native toxins Cry1Ab and Cry1Ac [22-26]. Compared with Cry1Ab and Cry1Ac, both Cry1AbMod and Cry1AcMod lack 56 amino acids at their N terminus, including all of helix α-1 of domain I [22,26]. Although these modified toxins have not been commercialized yet, transgenic tobacco plants producing Cry1AbMod killed larvae of *Manduca sexta* that had reduced susceptibility to Cry1Ab caused by cadherin silencing mediated via RNA interference [25]. In previous work, however, the efficacy of Cry1AbMod and Cry1AcMod has not been reported for strains with documented resistance to Cry2Ab. Moreover, scientists have proposed using "pyramids" that combine the modified toxins with native toxins [26,27], but previous studies have not determined if antagonistic interactions among toxins would limit the efficacy of such combinations.

 Here we tested Cry1AMod toxins singly and in combinations with native toxins against pink bollworm (*Pectinophora gossypiella*), a major pest of cotton [28]. Field-evolved resistance to Cry1Ac in this invasive pest is associated with reduced efficacy of Bt cotton producing Cry1Ac in India [15,29], whereas its resistance to Cry1Ac has increased less in China [30] and not at all in the United States despite more than a decade of extensive exposure [31,32]. Previous results showed that Cry1AbMod and Cry1AcMod were effective against the AZP-R strain of pink bollworm, which is highly resistant to Cry1Ab and Cry1Ac but susceptible to Cry2Ab [22,33,34]. In this study, we evaluated pink bollworm larvae from a susceptible strain and the BX-R strain of pink bollworm, which is highly resistant to Cry2Ab as well as Cry1Ac and Cry1Ab [34]. The results show that Cry1AbMod and Cry1AcMod were effective against the resistant BX-R strain. In addition, tests with both the resistant and susceptible strains showed slight, but consistent synergism between Cry1AbMod and Cry2Ab, whereas the other combinations of toxins did not show consistent synergism or antagonism.

## Results

### Single Toxins

 The BX-R strain of pink bollworm, which we selected in the laboratory with Cry1Ac and Cry2Ab, was highly resistant to native Bt toxins, but not to Cry1AbMod and Cry1AcMod (Table 1). We calculated the resistance ratio as the concentration of toxin killing 50% of larvae (LC_50_) for the resistant strain divided by the LC_50_ for a simultaneously tested susceptible strain (APHIS-S). The resistance ratios were 210 for Cry2Ab, 270 for Cry1Ab, and 310 for Cry1Ac, but only 1.6 for Cry1AbMod and 2.1 for Cry1AcMod (Table 1).

**Table 1:** Efficacy of native Bt toxins Cry2Ab, Cry1Ab, Cry1Ac and genetically modified Bt toxins Cry1AbMod, and Cry1AcMod against a resistant strain (BX-R) and a susceptible strain (APHIS-S) of pink bollworm.

Strain	Toxin	n	Slope (SE)^a^	LC_50_ (95% FL)^b^	RR^c^	Survival (%)^d^
BX-R	Cry2Ab	600	0.94 (0.2)	90.3 (50 - 180)	210	81
	Cry1Ab	240	NA^e^	28.1 (NA)	27	100
	Cry1Ac	720	3.8 (0.7)	113 (82-150)	310	88
	Cry1AbMod	414	3.4 (0.5)	1.67 (1.1-2.3)	1.6	0
	Cry1AcMod	720	4.2 (1.2)	2.63 (1.6-3.2)^f^	2.1	0
APHIS-S	Cry2Ab	480	2.4 (0.4)	0.438 (0.21-0.65)	1.0	0
	Cry1Ab	240	3.9 (1.1)	0.103 (0.060-0.14)	1.0	0
	Cry1Ac	600	3.1 (0.5)	0.363 (0.27-0.45)	1.0	0
	Cry1AbMod	390	2.3 (0.4)	1.07 (0.43-1.7)	1.0	0
	Cry1AcMod	720	1.9 (0.3)	1.24 (0.093-2.3)^f^	1.0	0

a Slope of the concentration-mortality line with its standard error in parentheses

b Concentration killing 50% with 95% fiducial limits in parentheses, in µg toxin per ml diet.

c Resistance ratio, the LC_50_ for a strain divided by the LC_50_ for APHIS-S for the same toxin.

d Survival at 10 µg toxin per ml diet adjusted for control mortality, n = 40 to 120 (mean = 82) larvae for each estimate.

e Not available

f 90% fiducial limits, 95% fiducial limits not available

 Consistent with the results from LC_50_ values summarized above, responses to a diagnostic concentration of each toxin show that the BX-R strain was highly resistant to the three native toxins tested, but not to the two modified toxins (Table 1). For the native toxins Cry1Ab, Cry1Ac and Cry2Ab tested at 10 µg toxin per ml diet, survival was 81 to 100% for the resistant strain compared with 0% for the susceptible strain (Table 1). In contrast, for the modified toxins Cry1AbMod and Cry1AcMod tested at the same concentration, survival was 0% in all trials for the both the resistant and susceptible strains.

 Evaluation of potency, which is inversely related to LC_50,_ showed that against the resistant strain, the modified toxins were more potent than the native toxins (Table 2). Against the susceptible strain, however, the modified toxins were less potent than the native toxins (Table 2). Using the data from Table 1, we calculated the potency ratio [26] for modified toxins relative to native toxins as the LC_50_ of a native toxin divided by the LC_50_ for a modified toxin. Against the resistant strain, the modified toxins were 11 to 68 times more potent than the native toxins (Table 2). For example, Cry1AcMod was 43 times more potent than Cry1Ac (Table 2). Conversely, against the susceptible strain, Cry1AbMod and Cry1AcMod were less potent than Cry1Ab, Cry1Ac, and Cry2Ab (potency ratio = 0.083 to 0.41, Table 2). 

**Table 2:** Potency of modified Bt toxins relative to native Bt toxins against resistant and susceptible strains of pink bollworm.

	Potency ratio for modified toxin relative to native toxin^a^	
Toxin pair	Resistant strain (BX-R)	Susceptible strain (APHIS-S)
Cry1AbMod/Cry1Ab	17	0.096
Cry1AcMod/Cry1Ac	43	0.29
Cry1AbMod/Cry1Ac	68	0.34
Cry1AcMod/Cry1Ab	11	0.083
Cry1AbMod/Cry2Ab	54	0.41
Cry1AcMod/Cry2Ab	34	0.35

a LC_50_ of a native toxin divided by the LC_50_ of a modified toxin (based on data from Table 1). Potency ratios > 1 indicate the modified toxin was more potent than the native toxin; potency ratios <1 indicate the modified toxin was less potent than the native toxin.

### Toxin Combinations

 The results indicate slight, but consistent synergism between Cry1AbMod and Cry2Ab against the resistant and susceptible strains of pink bollworm (Tables 3 and 4). For all eight combinations of concentrations of Cry1AbMod and Cry2Ab tested against both strains, the observed mortality was numerically higher than the mortality expected based on the responses to each toxin tested singly (Tables 3 and 4). Across the four combinations of Cry1AbMod and Cry2Ab tested against each strain, the mean increase in observed mortality relative to expected mortality was 19% for the resistant strain and 12% for the susceptible strain (Fisher’s exact test, P = 0.0003 and 0.002, respectively; Tables 3 and 4). 

**Table 3:** Efficacy of Bt toxins Cry1AbMod, Cry1Ac and Cry2Ab singly and in combinations against a resistant strain (BX-R) of pink bollworm (see Methods for details).

	Concentration	Mortality (%)^a^			
Toxin set	(µg per ml diet)	Obs^b^	Exp^c^	Obs - Exp^d^	*P* ^e^
Cry1AbMod	0.1	12	—	—	—
	1.0	27	—	—	—
Cry1Ac	30	12	—	—	—
	100	35	—	—	—
Cry2Ab	3.0	4	—	—	—
	10	8	—	—	—
Cry1AbMod + Cry1Ac	0.1 + 30	0	22	-22	0.004
	0.1 + 100	42	42	0	1.0
	1.0 + 30	46	35	11	0.37
	1.0 + 100	81	52	29	0.02
	Mean	42	38	4	0.36
Cry1AbMod + Cry2Ab	0.1 + 3.0	23	15	8	0.24
	0.1 + 10	42	18	24	0.03
	1.0 + 3.0	46	30	16	0.16
	1.0 + 10	62	33	29	0.02
	Mean	43	24	19	0.0003
Cry1Ac + Cry2Ab	30 + 3.0	20	15	5	0.54
Cry1AbMod + Cry1Ac + Cry2Ab	0.1 + 30 + 3.0	27	25	2	0.80
	1.0 + 30 + 3.0	63	38	25	0.02
	Mean	45	31	14	0.10

a All mortality values are adjusted for control mortality.

b Observed mortality

c Expected mortality for combinations of two or three toxins

d Observed mortality - expected mortality; synergism causes positive values and antagonism causes negative values

e Probability that the difference between observed and expected mortality occurred by chance based on Fisher’s exact test

**Table 4:** Efficacy of Bt toxins Cry1AbMod, Cry1Ac and Cry2Ab singly and in combinations against a susceptible strain of pink bollworm (APHIS-S) (see Methods for details).

	Concentration	Mortality (%)^a^			
Toxin set	(µg per ml diet)	Obs^b^	Exp^c^	Obs - Exp^d^	*P* ^e^
Cry1AbMod	0.1	4	—	—	—
	1.0	45	—	—	—
Cry1Ac	0.03	7	—	—	—
	0.1	4	—	—	—
Cry2Ab	0.1	16	—	—	—
	0.3	53	—	—	—
Cry1AbMod + Cry1Ac	0.1 + 0.03	11	11	0	1.0
	0.1 + 0.1	20	7	13	0.01
	1.0 + 0.03	44	49	-6	0.52
	1.0 + 0.1	53	47	5	0.53
	Mean	32	29	3	0.39
Cry1AbMod + Cry2Ab	0.1 + 0.1	44	19	25	0.0004
	0.1 + 0.3	64	55	9	0.27
	1.0 + 0.1	56	54	2	0.87
	1.0 + 0.3	87	74	13	0.06
	Mean	63	51	12	0.002
Cry1Ac + Cry2Ab	0.03 + 0.1	22	22	0	1.0
Cry1AbMod + Cry1Ac + Cry2Ab	0.1 + 0.03 + 0.1	22	25	-3	0.71
	1.0 + 0.03 + 0.1	59	58	1	1.0
	Mean	40	41	-1	0.91

a All mortality values are adjusted for control mortality.

b Observed mortality

c Expected mortality for combinations of two or three toxins

d Observed mortality - expected mortality; synergism causes positive values and antagonism causes negative values

e Probability that the difference between observed and expected mortality occurred by chance based on Fisher’s exact test

 In contrast, for both strains, no significant overall difference between observed and expected mortality occurred when the data from all concentrations tested were pooled for each of the other three toxin combinations: Cry1AbMod + Cry1Ac, Cry2Ab + Cry1Ac, or Cry1AbMod + Cry1Ac + Cry2Ab (Tables 3 and 4). Thus, we detected no consistent synergism or antagonism in these three combinations.

 Considering each of the 22 comparisons for observed versus expected mortality for each set of toxin concentrations tested, statistically significant synergism was indicated in six cases and statistically significant antagonism in one case (Tables 3 and 4). Three of the six cases showing synergism occurred with Cry1AbMod + Cry2Ab and contributed to the overall significant synergism of this combination for each strain. The other three cases of synergism consist of one case for Cry1AbMod + Cry1Ac for each strain and one case for the trio of toxins tested against the resistant strain (Table 3).

 The single case of apparent antagonism involved the lowest concentrations tested against the resistant strain (1 µg Cry1AbMod per ml diet + 1 µg Cry1Ac per ml diet) with observed mortality = 0% and expected mortality = 22% (Table 3, *P* = 0.004). Despite the statistical significance of this result, we suspect it has little biological significance because no significant antagonism was observed with the other seven sets of concentrations of these two toxins tested against the resistant and susceptible strains (Tables 3 and 4).

## Discussion

 The results here with the BX-R strain of pink bollworm are the first showing efficacy of Cry1AbMod and Cry1AcMod against an insect strain highly resistant to Cry2Ab as well as to Cry1Ab and Cry1Ac. Based on the results reported here and previously, the potency of at least one of the two modified toxins was higher than its native counterpart against 7 of 11 resistant strains tested [22,23,26,35]. These seven resistant strains represent six species from four families of Lepidoptera: the AZP-R and BX-R strains of *P. gossypiella* (Gelechiidae); the NO-QAGE strain of *Plutella xylostella* (Plutellidae); the GipBtR strain of *Trichoplusia ni* and the YHD3 strain of *Heliothis virescens* (Noctuidae); and the KS strain of *Ostrinia nubilalis* and the Bt-RR strain of *Diatraea saccharalis* (Crambidae) [22,23,26]. 

 The results suggest it is unlikely that antagonistic interactions would reduce the effectiveness of Cry1AbMod used in combination with native toxins Cry1Ac, Cry2Ab, or both against pink bollworm (Tables 3 and 4). Only one of 20 comparisons between observed and expected mortality for combinations of Cry1AbMod with native toxins showed significant antagonism (Tables 3 and 4). This lone case of statistically significant antagonism occurred only at the lowest concentrations of Cry1AbMod + Cry1Ac tested against the resistant strain. These results suggest that this potential antagonism could be avoided with high concentrations of either toxin. 

 The synergism between Cry1AbMod and Cry2Ab, which occurred for both the resistant and susceptible strains of pink bollworm, is the only consistent deviation from independent action of the toxins tested in combinations (Tables 3 and 4). The synergy between Cry1AbMod and Cry2Ab was modest, with a mean increase in observed mortality relative to expected mortality of 12% for the susceptible strain and 19% for the resistant strain (Tables 3 and 4). Stronger synergy between Bt toxins occurs in some other cases, such as between cytolytic (Cyt) toxins and Cry toxins against mosquitoes [36,37]. Whereas Cyt toxins can act as receptors for Cry toxins [38], we do not know the mechanism of the relatively weak synergy between Cry1AbMod and Cry2Ab. 

 Because the Cry1AbMod preparation included spores (Methods), we cannot exclude the possibility that spores contributed to the synergism between Cry1AbMod and Cry2Ab [39]. However, the tests of Cry1AbMod alone also included spores. Thus, any contribution of spores to the synergy between Cry1AbMod and Cry2Ab seen here would be limited to synergy between spores and the mixture of the two toxins that exceeded any synergy between Cry1AbMod and the spores. In addition, spores were also present in the tests with Cry1AbMod and Cry1Ac in which no consistent synergism occurred. More generally, we do not know if the responses of larvae to combinations of toxins in plants would match the responses observed in diet bioassays. 

 Consistent with previous results [22,23,26,35], the LC_50_ values here show that against a susceptible strain of pink bollworm, the modified toxins were less potent than native toxins Cry1Ab and Cry1Ac. Against the susceptible strain in this study, the potency ratio was 0.096 for Cry1AbMod relative to Cry1Ab and 0.34 for Cry1AcMod relative to Cry1Ac (Table 2). However, potency ratios <1 for the modified toxins relative to native toxins against susceptible strains do not necessarily signify a major drawback, particularly if the modified toxins are used jointly with native toxins (Tables 3 and 4). For example, the vegetative insecticidal Bt protein Vip3A is expected to be useful against *Helicoverpa armigera* in pyramided Bt cotton plants, despite a potency ratio of Vip3A relative to Cry1Ac of only 0.01 to 0.02 against susceptible strains of this pest [40,41]. Ultimately the value of a toxin in transgenic plants hinges not on its potency relative to other toxins, but its ability to kill the target pests when produced by the plants. In addition, the efficacy of different toxins produced by transgenic plants depends on their relative concentrations in the plants. Interactions among Bt toxins and plant allelochemicals can also affect efficacy.

 Progress in evaluating the potential utility of Cry1AbMod and Cry1AcMod now includes demonstration of their efficacy in diet bioassays when tested singly against seven strains of six major pests with resistance to one or more of the native toxins Cry1Ab, Cry1Ac, and Cry2Ab (Tables 1 and 2) [22,23,26] and in combination with Cry1Ac and Cry2Ab against resistant and susceptible strains of pink bollworm (Tables 3 and 4). In addition, Cry1AbMod added to diet or produced by transgenic tobacco plants was effective against larvae of a seventh species (*M. sexta*) in which susceptibility to CryAb was greatly diminished by silencing of the cadherin gene via RNA interference [25]. 

 Overall, the results suggest that the modified toxins might be useful in pyramids with native Bt toxins. In principle, such pyramids would be more effective than many current multi-toxin Bt plants that include a toxin to which pests have already evolved resistance in the field [8,15]. However, it remains to be determined if Cry1AbMod and Cry1AcMod will be useful against pink bollworm or other pests in the field. In any case, pests can probably adapt to modified Bt toxins used alone or in combination with other toxins. Nonetheless, along with other control tactics [31,42,43] and toxins that have been used less extensively than native Cry1A toxins [40,41,44,45], the modified toxins may broaden the options for managing some pests.

## Materials and Methods

### Insect Strains

We used two strains of pink bollworm: APHIS-S (susceptible) and BX-R (resistant). APHIS-S originated from Arizona and had been reared in the laboratory for >20 years without exposure to Bt toxins [46]. BX-R was started in December 2006 by pooling 875 pupae from two strains (BX-R1 and BX-R2) that had been selected in the laboratory for resistance to Cry2Ab [34]. 

### Toxins

 We used the protoxin form of five Bt toxins: Cry2Ab, Cry1Ac, Cry1Ab, Cry1AbMod and Cry1AcMod. Cry2Ab was produced in *E. coli* transformed with the plasmid pMP156 containing the *cry2Ab* gene from strain HD-1 of Bt subsp. *kurstaki* [47]. The source of Cry1Ac was MVP II, a liquid formulation obtained from Dow Agrosciences containing protoxin encapsulated in *Pseudomonas fluorescens* [48]. Cry1Ab, Cry1AbMod and Cry1AcMod were produced as suspensions containing protoxin and spores as described previously [22]. We tailored *cry1Ab* and *cry1Ac* genes to create the modified genes *cry1AbMod* and *cry1AcMod* using a three-step PCR process [22]. Based on the coding sequences, Cry1AbMod and Cry1AcMod proteins are expected to lack 56 amino acids at the N terminus compared with Cry1Ab and Cry1Ac. In addition to lacking all of the amino acids of helix α-1 of domain I, Cry1AbMod and Cry1AcMod lack four of the ten amino acids of helix α-2a (52-GAGF-55) and have two amino acid substitutions in helix α-2a (57-58VL changed to MA) to provide a methionine for translation. As expected, the weight of the protoxins was approximately 130 kD for Cry1Ab and 125 kD for Cry1AbMod and Cry1AcMod.

### Rearing, Bioassays and Selection

 Rearing, bioassays, and selection were done at 28 ± 2°C and 16 h light: 8 h dark with larvae fed wheat germ diet [48,49]. For bioassays and selection, we put neonates individually on diet into which toxin was incorporated [48,49]. After 21 days, we scored live fourth instars, pupae and adults as survivors [48,49]. 

 We selected BX-R for resistance by continuing the strain with survivors that fed on diet with toxin incorporated. From July 2007 to May 2009, BX-R was selected seven times with 3 to 100 µg Cry2Ab per ml diet. BX-R was selected with 10 µg Cry1Ac per ml diet in October 2009, 10 µg Cry2Ab per ml diet in January 2010, and 10 µg Cry1Ac + 3 µg Cry2Ab per ml diet in February 2010. From March 2010 to October 2011, BX-R was selected seven times (every two to four generations) with 10 µg Cry1Ac + 10 µg Cry2Ab per ml diet. Survival (adjusted for control mortality) during these seven selections ranged from 63 to 100% (mean = 78%).

### Single Toxins: LC_50,_ Resistance Ratio, Diagnostic Concentration and Potency Ratio

 To estimate the concentration of each toxin killing 50% of larvae (LC_50_), we used bioassays with at least three replicates in which five to eight concentrations (including 0 as a control) of each toxin were tested against a total of 24 to 60 larvae per concentration from each strain in each trial from July 2010 to August 2011. Our primary goal was to evaluate responses to the two modified toxins (Cry1AbMod and Cry1AcMod) and to the two toxins we used to select for resistance in BX-R (Cry1Ac and Cry2Ab). Thus, for both strains, we tested each of these four toxins in at least two trials conducted on separate dates. As a secondary goal, we evaluated responses to Cry1Ab to determine if BX-R was cross-resistant to this toxin. Because this was a secondary goal, we tested Cry1Ab against both strains only on one date. We analyzed the bioassay data for each toxin tested singly against each strain with the POLO program [50] to estimate the LC_50_ and its 95% fiducial limits. LC_50_ values with non-overlapping 95% fiducial limits are significantly different.

 We calculated resistance ratios as the LC_50_ of a strain divided by the LC_50_ of the susceptible APHIS-S strain tested simultaneously. We also calculated adjusted mortality at a diagnostic concentration (10 µg toxin per ml diet) as: 100% minus adjusted survival, where adjusted survival equals [survival (%) on treated diet divided by survival (%) on diet without toxin] X 100%. Because potency is inversely related to LC_50_, we calculated the potency ratio for modified toxins relative to native toxins as the LC_50_ of a native toxin divided by the LC_50_ for a modified toxin [26]. We report resistance ratios and potency ratios rounded to two significant digits. 

### Toxin Combinations

 For each insect strain, we used the bioassay method described above to test Cry1AbMod, Cry1Ac, and Cry2Ab singly, in pairs, and in a trio, with an aggregate total of 18 sets of concentrations including the control with untreated diet (Tables 3 and 4). Our primary goal was to evaluate two interactions: 1) between Cry1AbMod and Cry1Ac, and 2) between Cry1AbMod and Cry2Ab. Thus, we tested four sets of concentrations for each of these two toxin combinations against both the resistant and susceptible strains.

 We evaluated potential antagonism or synergism by testing for deviation from the null hypothesis of simple independent action [51], which assumes the proportion of larvae surviving exposure to a combination of toxins is the product of the proportions of larvae that survive exposure to each of the toxins separately [51]. For example, with two toxins:

(1) S_(ab)EXP_ = S_(a)OBS_ X S_(b)OBS_

where S_(ab)EXP_ is the proportion of larvae expected to survive exposure to a combination of toxins *a* and *b*, S_(a)OBS_ is the observed proportion of larvae that survived exposure to toxin *a*, and S_(b)OBS_ is the observed proportion of larvae that survived exposure to toxin *b*. We calculated expected mortality for larvae exposed to the combination of toxins *a* and *b* as (1 - S_(ab)EXP_) X 100%. We applied the same approach to test for synergism among three toxins: 

(2) S_(abc)EXP_ = S_(a)OBS_ X S_(b)OBS_ X S_(c)OBS_

where S_(abc)EXP_ is the proportion of larvae expected to survive exposure to a mixture of toxins *a*, *b*, and *c.*

 To calculate expected survival, we first calculated the observed adjusted survival for each toxin tested singly as survival on treated diet divided by survival on untreated diet (control). Survival on untreated diet ranged from 87 to 93% (mean = 90%). All of the results reported for treated diet are based on adjusted survival. 

 The susceptible strain was tested in February 2010 and both the susceptible and resistant strains were tested simultaneously in April 2010. On each date, the sample size was 30 larvae per concentration set for each strain for each of the 18 sets of concentrations (total sample size = 540 larvae per strain on each date). For the susceptible strain, the results for each of three toxins (Cry1AbMod, Cry1Ac and Cry2Ab) tested singly at each of two concentrations were similar between dates (range = 0 to 7% difference, mean difference = 2.2%) in five of six cases (3 toxins X 2 concentrations per toxin). For these five cases, we pooled the data across the two dates to calculate observed adjusted survival for each toxin tested singly. In the exceptional case, which was the susceptible strain tested at 1 µg Cry1AbMod per ml diet, adjusted survival was 50% in February 2010 vs. 89% in April 2010. The 89% survival is anomalously high, because survival ranged from 50 to 59% (mean = 55%) in the three other independent tests at this concentration (data from February 2010 collected as part of the test for interactions among toxins and data from Table 1 from July and September 2010 used to estimate the LC_50_ of Cry1AbMod). Accordingly, for this exceptional case, we used the mean adjusted observed survival (55%) from the three trials at this concentration conducted in February, July, and September 2010. This is a conservative approach for evaluating synergism, because the high survival (89%) in April 2010 would yield higher expected survival when Cry1AbMod was tested in combination with one or both of the other two toxins (Cry1Ac and Cry2Ab). By excluding the anomalously high survival estimate, we made it less likely that we would conclude synergism occurred in tests with Cry1AbMod and more likely that we would conclude antagonism occurred with tests with Cry1AbMod.

 We calculated the expected numbers of dead and live larvae by multiplying the expected mortality and survival rates, respectively, by the sample size used when each toxin was tested separately (sample size per toxin concentration per strain was 60 for APHIS-S and 30 for BX-R). For each of the 22 combinations of toxin concentrations tested (11 combinations tested per strain X 2 strains), we used Fisher's exact test (http://www.graphpad.com/quickcalcs/contingency1/) with 2-tailed probability to determine if a significant difference occurred between the observed and expected numbers of dead and live larvae. For each toxin combination tested against each strain, we also pooled the data across the different concentration sets before performing Fisher's exact test. For example, for BX-R versus Cry1AbMod + Cry1Ac, we pooled the data from the four concentration sets tested (n = 120 for observed response and n = 240 for expected response). This approach increases statistical power and determines if a consistent deviation from independent action occurred across the entire set of toxin concentrations tested for each strain for each toxin combination. 
